# The H_2_S-releasing naproxen derivative, ATB-346, inhibits alveolar bone loss and inflammation in rats with ligature-induced periodontitis

**DOI:** 10.1186/s13618-015-0025-3

**Published:** 2015-02-27

**Authors:** Bruno Schneider Herrera, Leila Santana Coimbra, Agatha Ribeiro da Silva, Simone Aparecida Teixeira, Soraia Katia Pereira Costa, John Lawrence Wallace, Luis Carlos Spolidorio, Marcelo Nicolas Muscara

**Affiliations:** Department of Pharmacology, Institute of Biomedical Sciences, University of Sao Paulo (USP), Sao Paulo, SP Brazil; Department of Physiology and Pathology, Araraquara School of Dentistry, Sao Paulo State University (UNESP), Araraquara, SP Brazil; Department of Pharmacology and Toxicology, University of Toronto, Toronto, ON Canada

**Keywords:** Periodontitis, Bone loss, ATB-346, H_2_S-releasing NSAID, Inflammation

## Abstract

**Background:**

In experimental periodontitis, non-steroidal antiinflammatory drugs (NSAIDs) effectively inhibit the resultant alveolar bone loss. However, their deleterious gastric effects, observed in both animals and humans, dramatically limit their long-term use. It has been proven that the addition of a hydrogen sulfide (H_2_S)-releasing moiety to classical NSAID structures results in antiinflammatory compounds with improved gastric safeness. In this way, we decided to compare the effects of naproxen with its H_2_S-releasing derivative ATB-346 on ligature-induced periodontitis in rats.

**Methods:**

Male Holtzman rats had a cotton ligature placed subgingivally around the lower right first molar during 7 days. During this period, groups of animals were daily treated with Na_2_S (a spontaneous H_2_S donor) or equimolar oral doses of naproxen (10 mg/kg) or ATB-346 (16 mg/kg). The mandibles were finally collected for histological analysis, radiographical measurements of alveolar bone loss and micro-computed tomography (μCT) analysis. Interleukin (IL)-1β, IL-6 and IL-10 were quantified in gingiva samples, and the stomachs were also collected for scoring of tissue damage and measurement of myeloperoxidase (MPO, a marker of granulocyte infiltration).

**Results:**

Ligature-induced bone loss was significantly inhibited by all the treatments, although only ATB-346 treatment resulted in significant inhibition of bone defect and other histological characteristics (such as flatness of the gingival epithelium, chronic inflammatory cell infiltration and loss of connective tissue in the gingival papillae). Both naproxen and ATB-346 inhibited the increase of gingival IL-1β and IL-6 secondary to periodontitis, but IL-10 was unaffected. Significant damage and increased MPO contents were only found in the stomachs of the naproxen-treated animals.

**Conclusion:**

The H_2_S-releasing moiety in the ATB-346 compound not only does not impair the effects of the parent naproxen on periodontitis, but also improves bone quality and prevents the gastric mucosa damage due to prostaglandin inhibition, thus configuring a potentially new adjuvant therapy for periodontal diseases.

## Introduction

Periodontitis is a chronic inflammatory disease and a major public health concern considering that that it is among the most prevalent human diseases [1]. It is triggered by bacterial biofilms and, with other periodontal diseases, comprise a unique and complex group of inflammatory conditions that result in the destruction of the supporting structures of the dentition [2]. While the etiology of periodontitis is bacterial, it is becoming clear that the pathogenesis of disease is mediated by the host response [3].

Some studies show that periodontitis is associated with other several inflammatory conditions, such as arthritis, type-II diabetes, preeclampsia, preterm low birth weight, and cardiovascular diseases [4-9]. Since the pathogenesis of periodontitis shares similar aspects with many other inflammatory diseases, animal models of the disease are recognized as valid tools for the study of effector cell mediated inflammation [10].

In inflamed periodontal tissues, cylooxygenase-2 (COX-2) expression is significantly up-regulated [11] and COX-2-derived prostaglandin E_2_ (PGE_2_), produced by activated macrophages and fibroblasts, is present in the crevicular fluid of patients with periodontitis and considered as one of the major inflammatory mediators of alveolar bone destruction [12,13]. In fact, alveolar bone loss secondary to ligature-induced periodontitis in rats is significantly reduced during treatment with either selective COX-2 inhibitors [14,15] or traditional non-selective NSAIDs [16-18]. In humans, PGE_2_ is associated with disease aggressiveness and can be used as a reliable indicator of current clinical periodontal destruction [19].

In this way, COX inhibition by NSAIDs would be an attractive clinical pharmacological approach aimed to reduce both PGE_2_ production at the site of inflammation and tissue destruction [14,15]. However, the chronic use of NSAIDs is limited by the well-known, and clinically relevant, renal and cardiovascular occurrences, in addition to gastrointestinal ulceration and bleeding [20,21].

In this way, new classes of NSAIDs capable of releasing nitric oxide (NO) [22] or H_2_S [23-27] *in vivo* have been developed, with basis on the ability of these mediators to effectively antagonize many of the deleterious side-effects of traditional NSAIDs. For example, several studies show that the new H_2_S-releasing NSAIDs, such as those derived from mesalamine [23,24], indometacin [27], diclofenac [26] or naproxen [25,28-30], present significant advantages over the parent NSAIDs, with no impact on cardiovascular system, decreased gastric effects, and even accelerated healing activity of pre-existing gastric ulcers [25].

Using a rat model of knee joint synovitis, we have recently shown that, despite the similar effects of both naproxen and its H_2_S-releasing derivative ATB-346 on inhibiting joint pain, edema and inflammatory cell recruitment to the knee-joint cavity, the H_2_S-releasing NSAID exerted no significant gastric damage, thus making of ATB-346 an interesting therapeutic alternative to traditional naproxen [30].

Regarding the relationship between H_2_S and periodontitis, published studies show discrepant results depending on the H_2_S donor employed, the administration route and doses [31-33].

Based on the considerations above, we thus decided to study the effects of the administration of Na_2_S (an H_2_S donor), naproxen and its H_2_S-releasing derivative ATB-346 in rats with ligature-induced periodontitis.

## Material and methods

### Animals

All the experimental protocols were approved by the local Ethics Committee for Animal Experimentation and performed in accordance with the guidelines of the Brazilian College for Animal Experimentation (COBEA). Male adult Holtzman rats (*Rattus norvergicus albinus*) weighing 250–300 g, were used in the experiments. During the length of the experimental protocol, the rats were kept in a quiet room with controlled temperature (22 ± 1°C), humidity (65-75%) and a 12-h light–dark cycle, and they were fed standard rat chow and received tap water *ad libitum*.

### Chemicals

All the drugs and reagents were purchased from Sigma-Aldrich (Brazil), unless otherwise stated.

### Induction of periodontitis, treatments and sample collection

The rats were anesthetized with ketamine (80 mg/kg, i.p.; Francotar, Virbac do Brasil Ind. e Com. Ltda, Brazil) and xylazine (16 mg/kg, i.p.; Kensol, Konig S.A., Brazil) and a 3–0 cotton ligature was placed subgingivally around the lower right first molar, as previously described [34]. Sham-operated animals had the ligature immediately removed after the procedure.

Following ligature placement, groups of animals started to receive daily oral doses of Na_2_S (dissolved in the drinking water at 45 mg/L, equivalent to a daily dose of approximately 10–12 mg/kg/day), equimolar doses of naproxen (10 mg/kg) or ATB-346 (16 mg/kg; Antibe Therapeutics Inc., Toronto, ON, Canada), or vehicle (1 ml/kg of 0.5% CMC). Treatments continued during the next 7 days, when the animals were euthanised.

Samples of gingiva surrounding the ligatured tooth were collected for measurement of cytokine contents, and the corpus region of the stomachs were also excised for assessment of macroscopical tissue damage and myeloperoxidase activity content (see below). The mandibles were removed for bone loss and histological analysis as shown below.

### Bone loss analysis

Digital X-ray images were also obtained for alveolar bone loss estimation by measurement of the distance between the cement-enamel junction (CEJ) and the alveolar bone crest at the mesial face of the ligatured tooth.

### Histological analysis

The madibles were kept in 10% formaldehyde solution during 48 h, followed by decalcification of the right hemi-mandibles in 4.13% EDTA (pH 7.2) at 4°C for approximately 3 months. Serial paraffin sections of 5 μm thickness were made on the mesial-distal aspects of the whole first lower right molars and stained with hematoxylin and eosin (H&E). Morphological studies were made on the buccal and lingual gingiva and alveolar bone.

### Micro-computed tomography (μCT) analysis

For quantitative and qualitative three-dimensional (3D) analysis of the alveolar bone defect, right hemi-mandibles of 5 rats per group were scanned dorso-ventrally using a microfocus X-ray CT system (Skyscan, Aartselaar, Belgium, 2003). The sagittal plane of the specimens was set parallel to the X-ray beam axis. The specimens were scanned at a resolution of 8.8 μm in all three spatial dimensions. The scans were gaussian-filtered and segmented using a multilevel global thresholding procedure for the segmentation of enamel, dentin, and bone [35]. CTan/CTvol software (Skyscan, Aartselaar, Belgium, 2003) was used for imaging and analysis. The volume of interest (VOI) was drawn with a slice-based method starting from the first slice containing the cement-enamel junction of the first molar and moving dorsally for 168 slices in the area 3 mm above the cement enamel junction. On the original 3D image, the microstructural variables, area and volume of bone defect, were calculated directly from the binarized VOI.

### Analysis of gingival cytokine contents

Interleukin (IL)-1β, IL-6 and IL-10 were measured in the collected gingiva samples by ELISA using commercial kits (R&D Systems Inc., Minneapolis, MN, USA) following the manufacturer instructions. Total protein contents were quantified according to the method described by Bradford et al. [36].

### Gastric damage assessment

At the end of the 7-day treatment period, the rats were euthanized and the stomachs were excised. The severity of damage was blindly scored as described in detail previously [37]. The length of each hemorrhagic erosion (in mm) was measured from the digitalized pictures using the software ImageJ (NIH, USA), and a gastric damage score was calculated by summing the lengths of all erosions in each stomach.

In addition, the activity of myeloperoxidase (MPO), a hemoprotein located in azurophilic granules of neutrophils, was used as a biochemical marker of neutrophil infiltration into the collected stomach samples, and was spectrophotometrically measured as previously described [38].

### Statistical analysis

All data are expressed as mean ± SEM. Differences among the group means were analyzed by one-way ANOVA followed by Tukey’s test for multiple comparisons. Values of P < 0,05 were considered as statistically significant.

## Results

### Ligature induced bone loss

**Figure 1:**
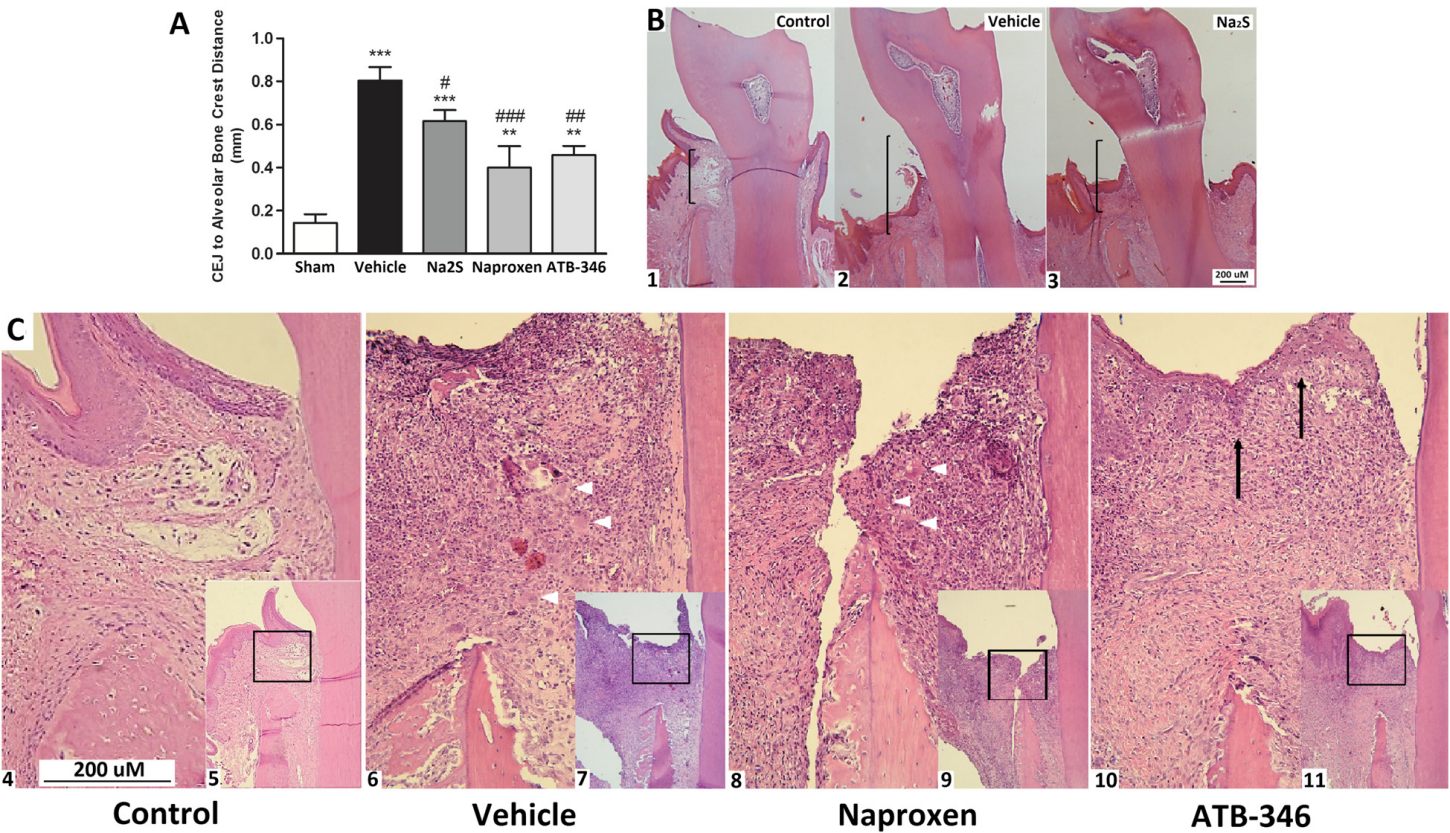
**Ligature-induced alveolar bone loss and the inflammatory parameters is reduced in rats treated with naproxen or its H**_**2**_ **S-releasing derivative ATB-346. Panel A**: Distance from the cemento-enamel junction (CEJ) to the alveolar bone crest analyzed by digital X-ray images (n = 8). **P < 0.01 and ***P < 0.001 vs. Sham; ^#^P < 0.05, ^##^P < 0.01 and ^###^P < 0.001 vs. Vehicle. Histological aspect of gingival tissues. **Panel B** (4 × magnification): 1. Control, 2. Vehicle, 3. Na_2_S; **panel C** (20 × magnification): 4. Control, 6. Vehicle, 8. Naproxen, 10. ATB-346 (the respective inserts 5, 7, 9 and 11 are at a 10 × magnification). Δ: Giant cells; →: markedly reduced cellular infiltrate, preserved collagen fibers and gingival epithelium in comparison with the other groups.

As shown in Figure 1 (panel A), after 7-day ligature, significant alveolar bone resorption was observed in the vehicle-treated animals in comparison with the Sham group, as radiographically assessed by measurement of the distances between the CEJ and the alveolar bone crest (0.80 ± 0.06 vs. 0.14 ± 0.04 mm, P < 0.001). Bone loss was significantly inhibited by all of the treatments (naproxen: 0.62 ± 0.05 mm, P < 0.05; ATB-346: 0.46 ± 0.04 mm, P < 0.01; Na_2_S: 0.4 ± 0.1 mm, P < 0.001).

### Histological analysis

Figure 1 also shows that normal morphology was observed in the Sham rat gingiva obtained from both the vestibular and lingual faces of the lower first molar. The oral, sulcular and junctional epithelium presented classical structures, and the connective tissue was dense, formed by thick collagen fibers, interspersed with delicate vessels and fibroblasts. Few inflammatory cells were found in the junctional epithelium region (panel B1).

The presence of a cotton ligature in a submarginal position around the lower first molar induced the classical microscopic characteristics of periodontitis, such as flatness of gingival epithelium, intense infiltration of chronic inflammatory cells, presence of giant cells, loss of connective tissue in the gingival papillae and evident alveolar bone resorption activity (panels B2 and C1). Na_2_S treated rats presented with evident decrease of the extension of chronic inflammation and partially conserved the connective tissue papillae, which is associated with milder bone resorption activity (panel B3). Naproxen treatment did not result in microscopic changes of the aspect of ligature-induced host response when compared with the vehicle-treated rats (panels B2 and C2). However, the administration of ATB-346 resulted in marked inhibition of the ligature-induced host response in the gingival tissues, characterized by the evident decrease of the extension of chronic inflammatory cells, giant cells and partially conserved connective tissue papillae, as indicated by the preservation of collagen fibers and gingival epithelium (panel C4).

### Bone defect area and volume values analyzed by μCT

**Figure 2:**
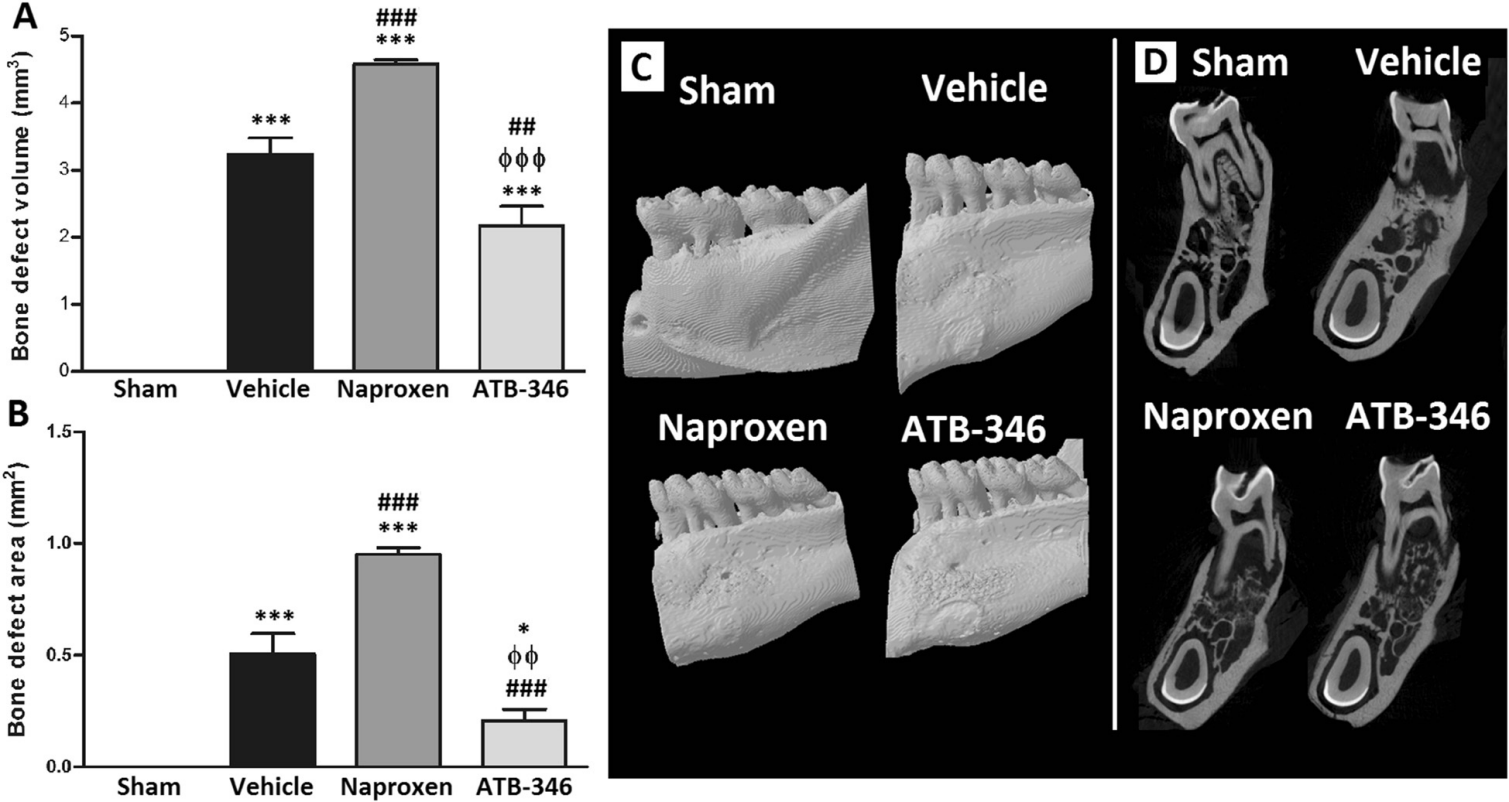
**Micro-computed tomography (μCT) shows that ATB-346 inhibited the increase of the bone area and volume secondary to ligature-induced periodontitis.** Bone analysis by μCT in terms of both area **(panel A)** and volume **(panel B)** of bone defect. *P < 0.05 and ***P < 0.001 vs. Sham; ^##^P < 0.01 and ^###^P < 0.001 vs. Vehicle; ^ϕϕ^P < 0.01 and ^ϕϕϕ^P < 0.001 vs. Naproxen **Panel C**: Representative three-dimensional reconstructions of μCT analysis performed on the left hemimandible of the animals subjected to periodontitis. **Panel D**: μCT images on the sagittal plane of the left hemimandible of the animals depicting the alveolar bone in the interradicular area (n = 6 for each experimental group).

In addition to the inflammation and bone loss caused by the presence of the ligature around the first molar during 7 days, significant defects were induced in bone area and volume (area: 0.50 ± 0.13 vs. 0 ± 0 mm^2^, P < 0.001; volume: 3.24 ± 0.24 vs. 0 ± 0 mm^3^, P < 0.001; Figure 2, panels A and B, respectively). Treatment of the animals with naproxen potentiated these ligature-induced defects (area: 0.95 ± 0.03 mm^2^, P < 0.001; volume: 4.58 ± 0.07 mm^3^, P < 0.001). However, these bone defect parameters were significantly reduced in the ATB-346-treated rats (area 0.21 ± 0.05 mm^2^, P < 0.001; volume: 2.17 ± 0.2907 mm^3^, P < 0.01).

### Gingival inflammatory cytokines

**Figure 3:**
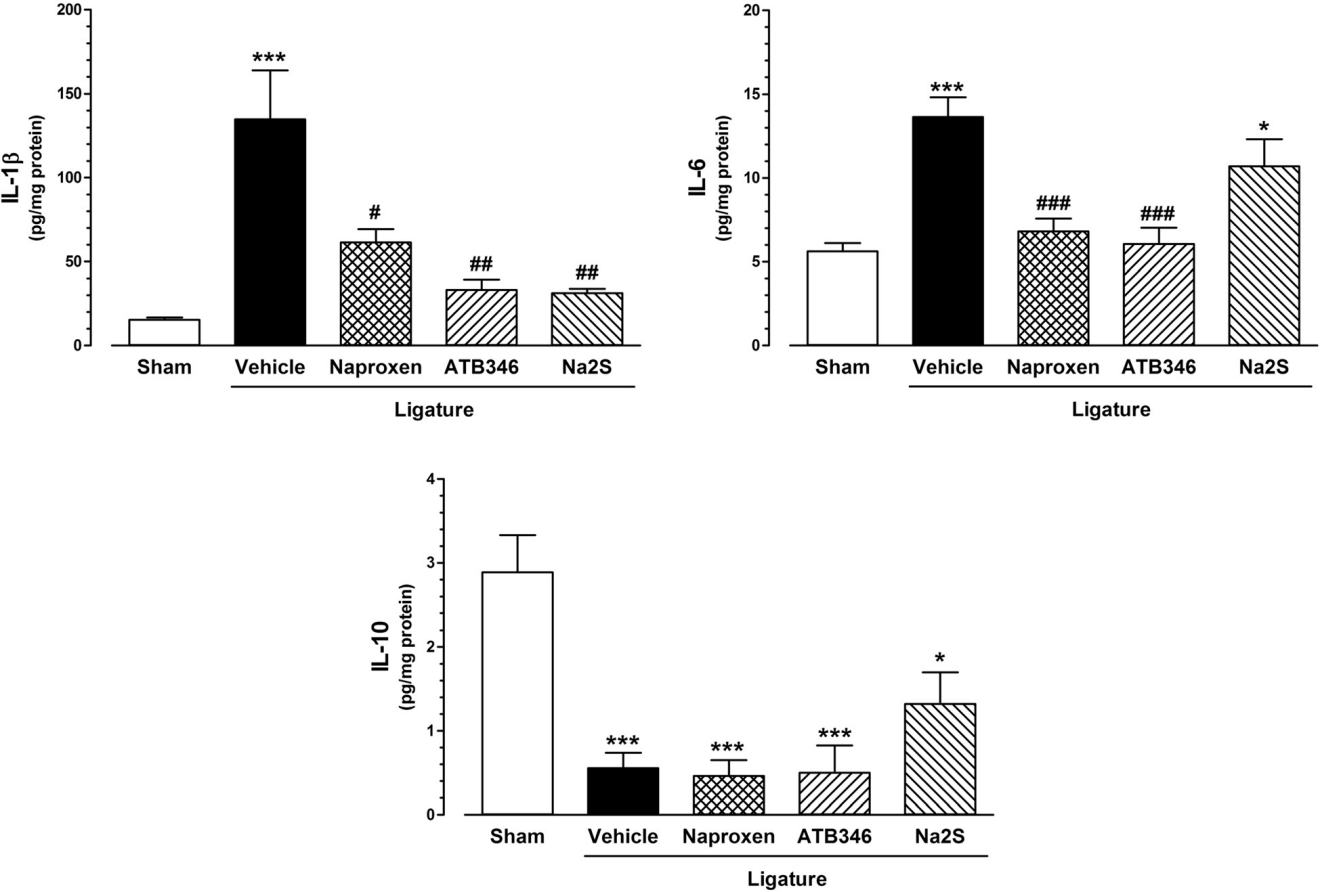
**Gingival IL-1β and IL-6 contents are increased in rats with ligature-induced periodontitis but counterregulated by treatment with either naproxen or ATB-346.** IL-1β, IL-6 and IL-10 contents measured in rat gingival samples obtained from the different treatment groups. *P < 0.05 and ***P < 0.001 vs. Sham; ^#^P < 0.05; ^##^P < 0.01 and ^###^P < 0.001 vs. Vehicle (n = 6 for each group).

As shown in Figure 3, the presence of ligature induced significant increases in the gingival contents of IL-1β and IL-6, as well as decreased IL-10, in comparison with the Sham animals (IL-1β: 134.8 ± 29.1 vs. 15.3 ± 1.5 pg/mg of protein; IL-6: 13.7 ± 1.2 vs. 5.6 ± 0.5 pg/mg of protein; IL-10: 0.6 ± 0.2 vs. 2.9 ± 0.4 pg/mg of protein; P < 0.001 for all the three cytokines). Treatment with either naproxen, ATB-346 or Na_2_S resulted in significant decrease of gingival IL-1β contents (61.4 ± 7.8, 33.3 ± 6.0 and 31.2 ± 2.6 pg/mg of protein, respectively; panel A), but only naproxen and ATB-346 resulted in decreased IL-6 (6.8 ± 0.8 and 6.1 ± 1.0, respectively; panel B). Regarding IL-10 contents, none of the treatments affected the ligature-induced decrease; panel C).

### Gastric effects

**Figure 4:**
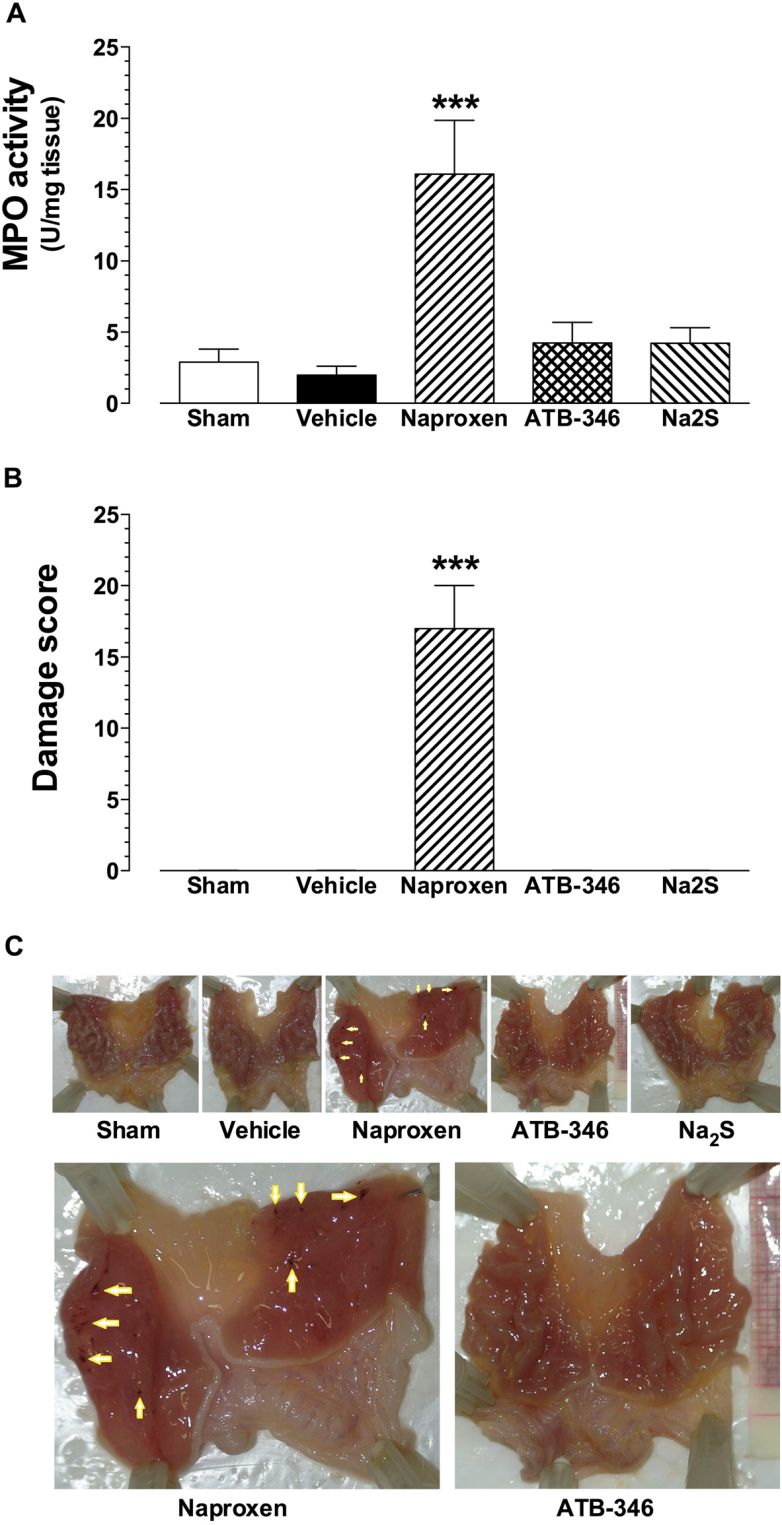
**Seven-day treatment with naproxen, but not ATB-346, induces gastric mucosa damage and increased myeloperoxidase activity (MPO).** MPO activity content **(panel A)** and tissue damage score **(panel B)**, determined in rat stomach samples obtained from the different treatment group. **Panel C**: Representative pictures of stomachs obtained from each experimental group (pictures corresponding to the naproxen and ATB-346 groups are also shown at higher magnification); yellow arrows indicate the presence of erosions. ***P < 0.001 vs. all the remaining experimental groups (n = 5 for each group).

As shown in Figure 4, the presence of ligature had no gastric effect, as assessed by either MPO activity (panel A) or macroscopical damage assessment (panels B and C). Only the naproxen-treated animals showed significantly increased gastric MPO activity (16.1 ± 3.8 U/mg tissue; P < 0.001 vs. all the remaining groups), which was paralleled by the gastric damage score (17 ± 3 vs. 0 for all the remaining groups; P < 0.001).

## Discussion

During the development of periodontal disease, bacteria and their products form a biofilm covering the tooth surface in the subgingival area, which can have direct deleterious effects on periodontal tissues [2]. In susceptible individuals, the host-mediated response to the pathogenic flora is subsequently responsible for the destruction of periodontal connective tissues, including alveolar bone [39,40].

The use of NSAIDs is one of the most widely used pharmacological tools to intervene and inhibit an inflammatory response, by antagonizing pro-inflammatory pathways and/or signaling, mainly dependent on COX-derived eicosanoids [41]. Although previous reports show that the NSAID treatment of rats with experimentally induced periodontitis leads to inhibition of periodontium inflammation and bone loss [14,15], NSAID therapy is not applicable for treatment of clinical periodontitis [42]. Unfortunately, and despite the magnitude of the clinical improvement is measurable, the need for prolonged therapy discourages the long-term use of NSAIDs in face of their severe cardiovascular, renal and gastric side effects [20,21].

One proposed strategy to avoid systemic complications and, at the same time, achieving high concentrations of the drug at the diseased site, has been the development of topical NSAID formulations (such as gels, toothpastes or mouth rinses) to be locally applied on a daily basis. However, evidence shows that just transient effects occur after the NSAID is withdrawn; in addition, no long-term or multicenter prospective clinical trials have been perfomed to date examining whether the therapeutic effects of topical NSAIDs can be retained on a long-term basis [16].

In this study, we show that the development of experimental periodontitis in rats was significantly inhibited by treatment of the animals with a new H_2_S-releasing naproxen derivative - ATB-346, as clearly evidenced by the diminished alveolar bone loss, defect volume and area. In addition, we also show that the intense infiltration of chronic inflammatory cells and loss of connective tissue in the gingival papilla secondary to the presence of periodontitis was inhibited not only by treatment with ATB-346, but also with the inorganic H_2_S donor Na_2_S, thus reflecting the importance that exogenous H_2_S has by itself in the control and progression of periodontal disease in rats. Furthermore, the stomachs from animals treated with ATB-346 did not show the increased neutrophil contents (i.e., MPO activity) or tissue damage observed in the stomachs from the naproxen treated animals, thus demonstrating clear beneficial effects of ATB-346 over the parent drug. Indeed, it is based on these gastric protective properties without loss of its antiinflammatory activity that has led ATB-346 to undergo a phase I clinical trial for safety and pharmacokinetic evaluations in healthy human subjects (Antibe Press Release, Oct. 6, 2014).

H_2_S is produced in virtually every organ system in the body [43,44], and some studies show that H_2_S contributes to the maintenance of tissue and organ integrity during inflammation by acting as resolution mediator [25,45,46]. Resolution of inflammation is an active, agonist-mediated and well-orchestrated process that drives the return to tissue homeostasis [47], and emerging evidence suggests that non-resolving inflammation is a critical underlying component of many prevalent chronic diseases, including periodontal disease [9]. In fact, El-Sharkawy et al. [48], showed that the adjunctive treatment of chronic periodontitis (i.e. scaling and root planning) with a daily dietary supplementation with omega-3 fatty acids and low-dose aspirin, increase the production a variety of pro-resolution lipid mediators, leading to a reduction in probing depths and a significant attachment gain, as assessed after 3- or 6-month treatment.

The observation that ATB-346 is capable of decreasing periodontal inflammation and also prevent the occurrence of deleterious effects on the gastric mucosa secondary to the naproxen moiety, can thus be explained, at least in part, by the release of H_2_S, which can induce a shift from a pro-inflammatory to a pro-resolution status, both locally and systemically (i.e., in the stomach and periodontium, respectively).

The mechanisms and pathways of H_2_S signaling are not yet completely understood and its regulation is so complex that new mechanisms are continuously discovered before the former mechanisms are fully understood [49]. For example, it has been shown that H_2_S can not only activate ATP-sensitive K^+^ (K_ATP_) channels (involved in vascular relaxation [50] and myocardial protection [51]), but also close T-type calcium channels (involved in visceral pain-like nociception and somatic hyperalgesia [52]), inhibit NF-κB signaling pathway (downregulating the expression of cytokines, chemokines, adhesion molecules and apoptosis [53]), inhibit cytochrome oxidase [54] and scavenge reactive oxygen and nitrogen species (thus inhibiting oxidative stress and regulating NO vascular effects [44]).

Regarding the effects of the administration of inorganic sulfide salts on periodontal tissues, a previous study from Irie et al. [31] shows that the topical application of 3 μmol NaHS into the gingival sulcus of the rat first molar induces a transient increase of osteoclast differentiation with up-regulation of RANKL expression in osteoblasts, thus allowing the authors to conclude that H_2_S may contribute to alveolar bone resorption through RANKL expression. However, it is worth noticing that in the referred study, the employed NaHS dose was the result of applying 3 μL of a 1 M NaHS solution, a concentration that more resembles H_2_S toxicology than the concentrations at which H_2_S occurs under physiological conditions.

On the other hand, more recent studies using inorganic sulfide doses within the μmol/kg/day range (as used in this work), or *in vitro* concentrations within the μM range (that usually occur in biological fluids), show results in line with ours, in terms of H_2_S inhibiting activity on bone resorption. For example, Toker et al. [33] showed that the systemic administration of NaHS at three different doses (14, 28 or 70 μmol/kg/day) had no impact on the alveolar bone loss, but a significantly lower number of osteoclasts was observed by the authors at the highest NaHS dose. Moreover, Lee et al. [55] showed that NaHS inhibited the differentiation of mouse bone marrow cells into multinucleated TRAP-positive osteoclasts *in vitro*, in addition to reducing the mRNA expression of molecules involved in both nicotine- and LPS-induced osteoclastogenesis (such as RANKL, OPG, M-CSF, MMP-9, TRAP, and cathepsin K), thus suggesting that H_2_S has a potential therapeutic value for treatment of bone diseases, such as periodontitis.

## Conclusions

Despite the well-known beneficial effects of NSAIDs during inflammatory conditions, their use as adjuvants in the treatment of clinical periodontitis is not applicable, as the need for prolonged therapy would expose the patient to the risk of severe cardiovascular, renal and gastric side effects. From the results shown in this study, we provide the first evidence that the presence of the H_2_S-releasing moiety in the ATB-346 compound structure not only inhibits bone loss and inflammation in experimental periodontits but, more importantly, it also prevents the occurrence of deleterious effects on the gastric mucosa due to prostaglandin inhibition. We thus believe that these findings could further encourage clinical studies looking for the impact of H_2_S-releasing NSAID compounds as potential adjuvants for classical periodontal treatment.

## Abbreviations

ANOVA: Analysis of variance
ATB-346: The H_2_S-releasing naproxen derivative [2-(6-methoxy- napthalen-2-yl)-propionic acid 4-thiocarbamoyl-phenyl ester]
CEJ: Cement-enamel junction
CMC: Carboxymethyl celulose
MPO: Myeloperoxidase
COBEA: Brazilian College for Animal Experimentation
COX-2: Type-2 ciclooxigenase
EDTA: Ethylenediamine tetraacetic acid
ERK: Extracellular signal-regulated kinase
H_2_S: Hydrogen sulfide
I.p: Intraperitoneal
IL-10: Interleukin 10
IL-1β: Interleukin 1β
IL-6: Interleukin 6
M-CSF: Macrophage colony-stimulating factor
MKP-1: Mitogen-activated protein kinase phosphatase-1
MMP-9: Matrix metallopeptidase 9
Na_2_S: Sodium sulfide
NaHS: Sodium hydrosulfide
NF-κB: Nuclear factor kappa B
NO: Nitric oxide
NSAID: Non-steroidal antiinflammatory drug
OPG: Osteoprotegerin
PGE_2_: Prostaglandin E_2_
PIP3K: Phosphatidylinositol 3-kinase
PKC: Protein kinase C
RANKL: Receptor activator of nuclear factor kappa-B ligand
SEM: Standard error of the mean
TRAP: Tartrate-resistant acid phosphatase
μCT: Micro-computed tomography
